# Profiling Cardiometabolic Health in Jordan: A Call to Action to Improve Cardiovascular Health

**DOI:** 10.7759/cureus.38488

**Published:** 2023-05-03

**Authors:** Khalid Sawalha, Reda Asad, Fuad Habash, Angel López-Candales

**Affiliations:** 1 Cardiometabolic Medicine, University of Missouri Kansas City, Kansas City, USA; 2 Endocrinology, Diabetes and Metabolism, University of Missouri Kansas City, Kansas City, USA; 3 Cardiac Electrophysiology, Baylor Heart and Vascular Hospital, Dallas, USA; 4 Cardiovascular Medicine, University of Missouri Kansas City, Kansas City, USA

**Keywords:** profiling cardiometabolic health in jordan, preventative cardiology, cardiovascular disease, jordan, cardiometabolic health

## Abstract

Over the past decades, Jordan has witnessed unprecedented growth in the prevalence of cardiometabolic diseases, with a crude prevalence of 48.2% in 2020, up from 38% in 2009. This is slightly higher than the reported prevalence of 40% in the US population. According to the latest World Health Organization report published in 2020, Jordan’s leading cause of mortality is cardiovascular diseases with a cause-specific mortality rate of 18.6%. Since the advancement of the healthcare system in Jordan took place in the early 2000s, Jordan has witnessed a major epidemiologic transition where the burden of infectious disease has decreased, but on the other hand, the burden of cardiometabolic disease has increased as well. For such a country with limited resources and healthcare infrastructure where two-thirds of its population is below the age of 30, this is alarming. This growth can be attributed to a complex interplay between genetic and lifestyle factors. Smoking, physical inactivity, obesity, and unhealthy diet are considered major public health problems in Jordan, as reported in 2007 by the Jordanian Behavioral Risk Factor Surveillance. We attempt to raise awareness through this review article, so healthcare providers in Jordan understand the magnitude of the issue, and appropriate steps are taken to reestablish screening and management guidelines pertaining to cardiometabolic diseases in Jordan.

## Introduction and background

The Hashemite Kingdom of Jordan

The Hashemite Kingdom of Jordan, commonly known as Jordan, is an Arab country located in the heart of the Middle East. It occupies an area of 89,000 km^2^ with an estimated population of 11 million inhabitants according to the Jordanian Department of Statistics in 2021 [1]. Jordan is considered a young country with about two-thirds of the country’s population under the age of 30 years old [2]. 

Administratively, Jordan is divided into north, central, and south regions with around 83% of the population residing in the central region [3]. Per the World Bank country classification, Jordan is considered an upper middle-income country with insufficient supplies of natural resources such as water and oil [4]. Despite this, Jordan prides itself as one of the best Arab countries in the healthcare system in terms of life expectancies at birth (72/74 in years with a male/female ratio), maternal mortality rates (19 per 100,000 live births), and infant and child mortality rates (23 per 1000 live births) [5]. This was also evident during the first wave of the coronavirus (COVID-19) pandemic in August 2020 when Jordan’s unique response in taking measures to slow the spread and lower mortality rates as the cumulative number of COVID-19 cases during the first stage of the pandemic was 1133 infected, 9 deaths, and 238 active cases [6].

Among Jordanians, there is a well-known quote:

“Even when you’re full, you can still always eat 40 more bites of food.”

This reflects the importance of generosity and hospitality and the significance of food in Jordanian culture. For example, breakfast in Jordan might consist of deeply fried Falafel, Hummus, Ful, and Pita bread. Coming to Jordan’s national specialty is the traditional Bedouin feast dish Mansaf (typically eaten by hand) made of lamb cooked in Jameed (a sauce of fermented dried yogurt) served with rice and fried nuts, a diet certainly rich in calories, sodium, carbohydrates, and fat, and to complicate matters, this is usually followed by eating a high sugar, fat, and cholesterol dessert known as Kunafeh.

Jordan’s background profile

According to a combined report generated in 2015 from the Government of Jordan, the United Nations Children’s Fund, and the United Nations Development Programme, over the past 10 years, Jordan has experienced success in pursuing structural reforms in education, and health as well as privatization and liberalization. Furthermore, the Government of Jordan has taken great strides in introducing social protection systems and reforming subsidies [7].

Unfortunately, the country still faces significant challenges, particularly due to vulnerabilities regarding energy import dependency and disruption of gas supplies from Egypt. To aggravate matters, significant tensions in the region from Iraq and Syria have weighed down the Jordanian economy, widening its trade deficit and weakening investor confidence. This is further compounded by high unemployment and significant dependency on Gulf economies [7].

Amid all inequalities, the last reported Gross Domestic Product per capita in Jordan in 2021 was at 3781.53 US dollars, equivalent to 30% percent of the world's average [8].

These effects can be translated into poorer quintiles with less educated heads of households. Furthermore, over half of the population under 15 years of age lives in the poorest two quintiles [7].

In turn, Jordan has achieved nearly universal coverage and good availability of water services, without differences across places of residence or income quintiles. Living conditions are quite evenly distributed and most importantly, access to educational and medical facilities is not much affected by residence or income quintile [7].

The Centers for Disease Control and Prevention have been working in Jordan since 1998, not only to build a workforce to improve the use of data at all levels of the Ministry of Health but also to strengthen health systems and develop surveillance for a variety of health risk areas [9].

Unfortunately, a growing national population and a significant influx of refugees from all over the region have created situations that challenge Jordan’s ability to ensure that everyone can access quality health care. To complicate matters even further, hospitals are overcrowded and the country faces challenges in retaining and appropriately deploying a skilled healthcare workforce [10].

Snapshot of the health situation in Jordan

By all accounts, noncommunicable diseases (NCDs) account for nearly 80% of total deaths in Jordan, with cardiovascular diseases (CVDs) being the most significant cause of death, accounting for 25% of deaths [11].

Based on Jordan’s National STEPwise Survey for NCD risk factors reported in 2019, there is a significant prevalence of hypertension (52%), diabetes (20%), and CVD (25%) risk among adults aged 45-69 years old. In response to these numbers, the Jordan Ministry of Health in close collaboration with the World Health Organization started the World Health Organization's HEARTS program, aimed at strengthening prevention and control measures to reduce CVD adverse outcomes from a primary care level perspective [11].

These initiatives included healthy lifestyle counseling, the establishment of evidence-based treatment protocols, means to facilitate access to medicines, diagnosis, and treatments, as well as an introduction to risk-based CVD management with appropriate follow-up monitoring [11].

Unfortunately, when the WHO conducted a follow-up assessment of the HEARTS program, significant gaps were uncovered. However, a strong commitment by the Ministry of Health has been undoubtedly critical in moving forward with this clinical program with a strong commitment to improving performance monitoring indicators [11].

Finally, the overall aim of this priority is strictly aimed at strengthening the overall response to CVD as this has been deemed to be a national health priority, considering the heavy burden of CVD. The successful implementation of the WHO HEARTS program is foreseen as a key factor in reforming health care [11].

## Review

Cardiometabolic diseases in Jordan

Cardiometabolic disease (CMD) is defined by the American College of Cardiology as an integrated cluster of individual risk factors to develop cardiovascular disease, primarily hypertension, insulin resistance, dyslipidemia, and central obesity.

In 2009, the reported prevalence of CMD in Jordan was assessed through a survey and reported as 38% [12]. In 2017, using the same population, Ajlouni et al. reported a crude prevalence of 48.2% [12]. This significant increase in CMD prevalence was shown to increase significantly with age in both sexes in which men aged 18 to 29 years old had 13.1% compared to 66.9% for those aged 60-69 years old while in women [8], it was 11.7% and 85.2%, respectively [12].

Further studies also reported significant abnormalities regarding individual components of CMD, including that the prevalence of obesity was 44.7% with higher rates in women than men (48.2% vs. 36.1) with abdominal obesity being the most prevalent abnormality. Data also suggested that low high-density lipoprotein (HDL) cholesterol with elevated triglyceride levels although notably high among men (41.8%); these levels were far worse in women (59.1%) [12-14]. Finally, the prevalence of high fasting blood glucose levels was 47.4% in men compared to 40% in women [12-14].

Notable lifestyle changes in Jordan from 2009 to 2017

Data from Al-Nsour and associates regarding the prevalence of selected chronic, NCDs risk factors in Jordan using the Jordan Behavioral Risk Factor Surveillance Survey revealed that most lifestyle habits among Jordanians, including smoking, physical inactivity, and poor diet choices were critically determinant to describe the poor choices made by this society [13].

Particularly relevant to the choices Jordanians made to compromise their CVD outcomes, it is important to highlight the high prevalence of smoking among males (50.4%) [14]. In the case of women, we must exercise caution when examining the reported smoking prevalence of only 9.1%, as it is believed that this number is a clear underestimation of the true prevalence, mostly driven by cultural restrictions that limit reporting smoking status among females [14].

The true reality is that despite the long battle against tobacco, Jordan is found to have one of the highest prevalence rates of tobacco use in the region as well as the world [15]. This is manifested by using tobacco, shisha or hookah, and electronic cigarettes [16].

Certainly, decision-makers in Jordan should reinforce the already established laws regarding the public smoking prohibition to avoid a future public health catastrophe. Changing the cultural norms in promoting smoking during occasions such as weddings, funerals, or even public spaces is also needed.

To compound the challenges, Jordanians face to reduce CVD adverse outcomes reflect their lifestyle choices regarding exercise. Data suggests that only 12.5% of Jordanian adults are reported to be physically active [17].

Obviously, lack of exercise would undoubtedly be related to a high reported age-standardized prevalence of obesity of 60.4% among men and 75.6% among women, up from 32.7% and 59.8% in 1998, respectively [18,19]. To make matters worse, a Jordanian's favorite cuisine is mainly composed of Mansaf (the traditional Jordanian dish), Shawarma, Falafel, and Kunafeh; a diet high in fat, cholesterol, and carbohydrates.

Obviously, these are definitive concerns and if true improvements regarding CVD outcomes are to be made, more concrete efforts need to be implemented, particularly when more than two-thirds of its population is under the age of 30 years.

What to do next?

“Prevention is better than cure”, first attributed to the Dutch philosopher Desiderius Erasmus in around 1500, is now a fundamental principle of our modern healthcare strategies. There is no better time than now to apply this concept. Starting at younger ages, limiting our exposure to such causative events that increase our risk of having CMD seems to be the “golden opportunity”.

Obviously based on the data presented, the first step requires tangible efforts to improve Jordanian lifestyle changes. These efforts start with the adoption of a healthier diet. Given our current knowledge of Jordanian cuisine, there is an urgent need to reduce the consumption of saturated fat, sodium, sweets, and added sugar while increasing the consumption of fruits, vegetables, whole grains, omega-3 fats, and fish.

For obvious reasons, the adoption of either the Dietary Approaches to Stop Hypertension (DASH) diet or the Mediterranean diet would be the next logical step. However, significant steps are needed to teach the entire country and to make their population understand not only the nutritional value of a good diet but its overall impact on prevention with the outlook of improving CVD health. Certainly, major efforts to teach these habits at younger ages and in schools would also be a good start for future generations [20].

Aside from efforts to establish healthier eating habits, the next logical step is to promote physical activity. One necessary piece in these efforts requires the help of the decision-makers in Jordan, not only in creating free-access gyms and running tracks but also in rewarding individuals for maintaining memberships and promoting programs that will reward those who lose weight and maintain a healthy lifestyle.

Current exercise guidelines, as recommended by the American Heart Association, include (a) getting at least 150 minutes per week of moderate-intensity aerobic activity or 75 minutes per week of vigorous aerobic activity, or a combination of both, preferably spread throughout the week; (b) Add moderate- to high-intensity muscle-strengthening activity (such as resistance or weights) on at least two days per week; (c) Spend less time sitting. Even light-intensity activity can offset some of the risks of being sedentary; (d) Gain even more benefits by being active for at least 300 minutes (5 hours) per week, and (e) Increase the amount and intensity gradually over time [21].

In addition, critical efforts need to be invested in healthcare campaigns to raise awareness of smoking effects on health and its relation to CMD. This should be accomplished by primary care physicians, as they should be the initial point of contact in providing education and counseling as well as preventing the initiation of tobacco use, particularly among school-aged children and adolescents. To promote this further, “clinicians should lead by example”.

Certainly, health ministry and government officials might consider increasing tobacco prices and taxes on these products as well as limiting the number of smoking designated areas while applying fines to aid the population in healthier choices.

Jordan’s reality meets hard-core data

In a study by Elhneiti et al., these investigators addressed the association between heart diseases and personal health behaviors, including physical exercise, body weight, and tobacco, by randomly recruiting patients from primary healthcare centers from all practices in the middle region in Jordan [22].

Patients were included if they had a working diagnosis of heart disease were 18 years and older and either visited the outpatient department within the community hospitals or attended primary healthcare centers [22]. They noted that simply adherence to behavioral advice given by nurses with regard to maintaining a healthy body weight and tobacco cessation should be given top priority in the prevention of heart disease. Furthermore, regular physical activity is certainly needed to prevent the development of heart disease [22].

In a more recent observational multicenter study conducted by Alsaud et al., which included 1449 individuals recruited from different cities in the South, Middle, and North of Jordan to examine the presence of six CVD risk factors, including hypertension, diabetes mellitus, cigarette smoking, dyslipidemia, obesity, and family history of premature CVD were assessed in every subject using a questionnaire [23].

In addition to these variables, demographic data, including age, gender, educational level, height, and weight, were also collected. Furthermore, blood pressure readings as well as random lipid profiles and blood sugar were also recorded. The Institutional Review Board of King Abdullah University Hospital approved this study [23].

After analyzing their data, the most notable was that dyslipidemia was the most prevalent CVD risk factor among Jordanians. They noted a higher prevalence of 74.6% of dyslipidemia among males, which usually starts at a very young age (early twenties) and continues to worsen with age progression. Furthermore, dyslipidemias worsen proportionally with weight gain. On a positive note, the prevalence of dyslipidemia decreased proportionally with the increase in educational level suggested relating inversely to work stress. Finally, 31.47% of patients with dyslipidemia were smokers [23]. Furthermore, they also noted that clustering of CVD risk factors was more common in men than in women with more than five risk factors in individuals aged 45-65 years compared with younger or older groups. Furthermore, a sedentary lifestyle, unhealthy dietary behavior, low socioeconomic level, regular alcohol consumption, urbanization, and pollution were also noted as modifiable risk factors [23].

In conclusion, based on the data obtained, mitigation of CVD morbidity and mortality in the Jordanian population can be attained by initiating primary CVD medical and behavioral interventions to control the CVDs modifiable risk factors [23].

Final recommendations based on the overwhelming evidence

Based on several lines of evidence, despite all national Jordanian efforts, not only the population is afflicted with an adverse CMD risk profile but also individuals are at greater CVD risk. Therefore, explicit steps need to be taken to improve CVD outcomes.

**Figure 1:**
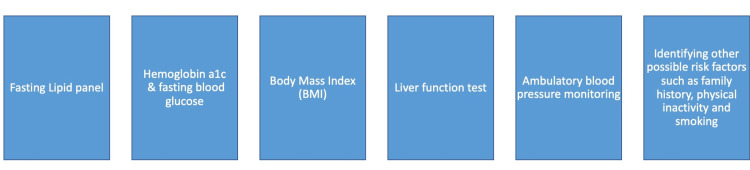
The recommended algorithm for screening for cardiometabolic diseases for adults > 18 years old BMI: Body Mass Index

Starting with the obvious, initial efforts based on the United States Preventive Services Task Force (USPSTF) recommended screening for every individual at high risk, particularly starting at a young age (Figure 1) [24]. For example, once an individual is identified with a body mass index (BMI) between 25 and 39.9, considered overweight or obese, four preventive health office visits should be allowed, along with unlimited nutritional counseling visits specifically for obesity per year and one set of recommended laboratory studies (fasting lipid profile, hemoglobin A1c, alanine transaminase and aspartate transaminase enzymes, and fasting glucose).

**Figure 2:**
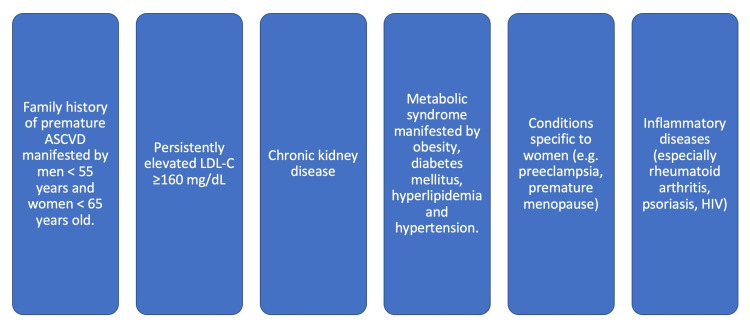
Current ASCVD risk enhancers ASCVD: Atherosclerosis Cardiovascular Disease. LDL-C: Low-Density lipoprotein - Calculated. HIV: Human Immune Deficiency.

Given the high prevalence of dyslipidemia, a fasting lipid profile should be performed at least once at the age of 18 years or older (Table 1) [25,26]. For patients with a normal screen before age 21 who remain at high risk, further risk assessments should be performed per the recommendations in Table 2. In addition, individuals with more than one risk enhancer should be considered at high risk (Figure 2).

**Table 1:** Showing the recommended cut-off values for the fasting lipid panel for adults aged 18 years or older LDL-C: Low-Density Lipoprotein - Calculated. HDL-C: High-Density Lipoprotein - Calculated. Mg/dl: Milligrams per deciliter.

Fasting Lipids	Normal	Abnormal	Special consideration
Total Cholesterol	<170 mg/dL	>200 mg/dL	
LDL-C	<100 mg/dL	>100 mg/dL	In patients with diabetes < 70 mg/dl. In patients with familial hypercholesterolemia refer to the prevention recommendations below
Triglyceride	<150 mg/dL	>150 mg/dL	
HDL-C	>45-60 mg/dL	<45 mg/dL	

**Table 2:** Showing primary prevention of cardiometabolic disease recommendations per age group (≤18, 19-39, and 40-75) Source: [20-22,25,26] LDL-C: Low-Density Lipoprotein - Calculated. ASCVD: Atherosclerosis Cardiovascular Disease. CT: Computed Tomography can. Mg/dl: Milligrams per deciliter.

Age group (Years)	Primary Prevention of Cardiometabolic Diseases Recommendations
≤18	Lifestyle changes implementation through dietary counseling, smoking cessation, and counseling. At least 150 minutes per week of moderate to vigorous activity. Screening for familial hypercholesteremia and if present consider statin.
19-39	In addition to prevention measures in ages 0 - 18: Screening for cardiometabolic diseases using Figure 1. If family history of premature atherosclerotic cardiovascular disease and LDL-C ≥ 160 mg/dl, consider statin.
40-75	In addition to prevention measures for ages 0 - 18: Use the ASCVD risk assessment tool to assess the 10-year ASCVD risk percent. Based on the risk percent, determine the need for statins. If the risk decision is uncertain, consider a coronary artery scan through CT.

For patients with a normal screen before age 21 who are not at high risk, screening for lipid abnormalities should then be repeated after the age of 35 for men and 45 for women. Hopefully, lifestyle modifications should have been already implemented.

Target values should be aimed as therapeutic goals: (a) total cholesterol of 170 mg/dL, (b) HDL-cholesterol of 50 mg/dL, (c) untreated systolic blood pressure of 110 mmHg, (d) no diabetes history (manifested by fasting glucose level of less than 100 mg/dl and Hba1c of less than 5.7%), and (e) nonsmoker [27,28].

We also recommend screening for high blood pressure in adults aged >18 years with ambulatory blood pressure monitoring or measurements outside of the clinical setting for diagnostic confirmation, those at high-risk, would-be patients with a mean daytime blood pressure of 130 to 139/80 to 89 mmHg, and those overweight or obese [29,30]. Adults aged 18 to 39 years without elevated blood pressure (ie, <130/80 mmHg) and without cardiovascular disease risk factors should be rescreened every three to five years [31,32].

The USPSTF recommends screening for abnormal blood glucose as part of cardiovascular risk assessment in adults aged 40 to 70 years who are overweight or obese. However, we should take into consideration starting at earlier ages, such as 18 years old, given that two-thirds of the Jordanian population is under the age of 30 years. Therefore, for adults >18 years old with hypertension or hyperlipidemia, as well as with a BMI ≥ 25 kg/m^2^, we suggest screening for type 2 diabetes as part of cardiovascular risk assessment [33]. Checking a hemoglobin A1C is a reasonable option, especially in patients with the highest risk of diabetes. Hba1c values less than 5.7% should be considered normal, 5.7-6.4% pre-diabetic, and 6.5% or higher as diabetic (Table 3) [28].

**Table 3:** The recommended cut-off values for fasting blood glucose and Hba1c for adults aged 18 years or older Hba1c: Glycated Hemoglobin.

Status	Fasting Blood Glucose	HbA1c
Normal	<100 mg/dL	<5.7%
Prediabetic	100-126 mg/dL	5.7-6.4%
Diabetic	>126 mg/dL	6.5% or more

With regards to adults who are above 40 years old, they should be screened for atherosclerotic cardiovascular disease using the Atherosclerotic Cardiovascular Disease 2013 Risk Calculator from the American Heart Association/American College of Cardiology and follow the recommendations regarding lifestyle changes and the use of statins (Table 4) [34].

**Table 4:** Indications for statins LDL-C: Low-Density Lipoprotein - Calculated. ASCVD: Atherosclerosis Cardiovascular Disease.

Statin Indications
If LDL-C ≥ 190 mg/dl at any age.
If a diagnosis of Familial hypercholesteremia is made.
If the patient is diabetic and of age 40-75 years old and LDL-C > 70 mg/dL.
If ASCVD risk percent is ≥ 7.5% or 5% -7.5% in the presence of risk enhancers as indicated in Figure 2.

Unquestionably, it takes “one step” to start; however, given the well-known resistance because of deficient social, cultural, economic, and governmental infrastructure, it would certainly take an inordinate effort to overcome a possible catastrophe. However, we should be optimistic that a change would happen if efforts were collaborated in the right way to envision any positive changes like those efforts seen in the first phase of the COVID-19 pandemic.

Therefore, we urge primary care physicians, pediatricians, internists, family medicine providers, and cardiologists to adopt the guidelines mentioned above and practice an active role in decreasing the CMD risk factors. In doing so, not only should appropriate healthy lifestyle counseling be instituted early on but also, be effective in conducting screening of these individuals, particularly if they are already afflicted by obesity, hypertension, hyperlipidemia, and diabetes.

These measures should be prioritized and taken to overcome any possible gain that ultimately results in any significant impact that can reduce CMD at a local level by redirecting local or national funds appropriately into effective programs that would be successfully productive. The health of Jordanians is unquestionably contingent on these accountable efforts.

On a final note, emphasizing the collaborative efforts between healthcare providers, decision-makers, and insurance companies in Jordan in facilitating sustainable preventative healthcare practices would be more advantageous for long-term public health and the economy.

## Conclusions

The road to achieving the dream of long-lasting CVD health improvement in Jordan is still long, with a strong need for more coordinated efforts of nongovernmental spending, more cardiac specialized centers of excellence, more efforts to integrate research with clinical practice, and a widespread population health literacy program. Steps in the right direction might get us to reach our goal of improving outcomes and reducing adverse events.
